# ﻿The green lacewing genus *Anachrysa* Hölzel, 1973 (Neuroptera, Chrysopidae): a new species, two new combinations, and updated key to species

**DOI:** 10.3897/zookeys.1106.83229

**Published:** 2022-06-16

**Authors:** Yunlong Ma

**Affiliations:** 1 Engineering Research Center for Forest and Grassland Disaster Prevention and Reduction, Mianyang Normal College, Mianxing West Road, 621000, Mianyang, China Engineering Research Center for Forest and Grassland Disaster Prevention and Reduction, Mianyang Normal College Mianyang China

**Keywords:** Key, new combinations, new species

## Abstract

A new species, *Anachrysaadamsi***sp. nov.**, from Yunnan, China is described. Two new combinations are proposed, namely *Anachrysaalviolata* (Yang & Yang, 1990), **comb. nov.** and *Anachrysatriangularis* (Yang & Wang, 1994), **comb. nov.** An updated key to species is also provided.

The paper has been retracted by the publisher due to evidences of misconduct.

The removed version is archived for the purpose of resolving nomenclatural questions and is available from the publisher upon request.

